# Impact of High-Fidelity Simulation on the Acquisition of Spiritual Competencies in a Nursing Degree in Spain: An Experimental Pre–Post Study

**DOI:** 10.1007/s10943-025-02285-8

**Published:** 2025-03-14

**Authors:** Jordina Domènech-Sorolla, Laura Martínez-Rodríguez, Sara Pedregosa-Fauste, Arnau Muns-Orenga, Fernando García-Díaz, Maria Dolores Fernández-Pascual

**Affiliations:** 1 Department of Nursing and Physiotherapy, University of Lleida, Lleida, Spain; 2 Grupo de Investigación Enfermera en Simulación en Catalunya y Andorra GRISCA, Barcelona, Spain; 3 Grupo de Innovación Docente D-CIDES-Fundación Índex, Barcelona, Spain; 4 Fundamental and Clinical Nursing Department, Faculty of Nursing, University of Barcelona, Barcelona, Spain; 5 Grupo de Innovación Docente INTERMASTER, Universitat de Barcelona, Barcelona, Spain; 6 Grupo de Innovación Docente IDhEA-Fundación Index, Barcelona, Spain; 7 Departamento de Filosofía, IES La Madraza, Granada, Spain; 8 Department of Health Psychology, Faculty of Health Sciences, University of Alicante, Alicante, Spain; 9 Instituto de Investigación Sanitaria y Biomédica de Alicante (ISABIAL), Alicante, Spain

**Keywords:** High-fidelity simulation, Spiritual care, Nursing education, Spiritual competencies, Experiential learning

## Abstract

High-fidelity simulation is an educational technique that utilizes advanced technology and realistic clinical scenarios to replicate real-life situations, offering students a safe and immersive environment to practice and enhance their skills. A quantitative experimental study with a pretest–posttest single-group design was conducted, where 143 first-year undergraduate nursing students (*n* = 143) participated in a simulation program focusing on spiritual care. The response rate was 49.5%, calculated as the number of students who completed the questionnaire (143) divided by the total number of students approached (289). The pretest and posttest were conducted one month apart. The Spirituality and Spiritual Care Rating Scale (SSCRS-Sp) was used to assess changes in students' spiritual competencies before and after the simulation. Results indicated significant improvements in students' perceptions of spiritual care, suggesting that simulation is an effective method for enhancing spiritual competencies.

## Introduction

Simulation is an experiential learning activity designed to immerse students in realistic scenarios that closely replicate the situations they may encounter in their professional careers (Gaba, 2004). The *International Nursing Association for Clinical Simulation and Learning* (INACSL) defines simulation-based experiential learning as an educational strategy that creates conditions imitating real-life situations that students may experience (Sittner et al., 2015). High-fidelity simulation involves the use of advanced technological simulators or standardized patients (trained actors) and aims to replicate authentic clinical environments to enhance the educational experience (Raurell-Torredà & Gómez-Ibañez, 2017; Wu et al., 2024). According to the INACSL Standards Committee, the structured process of high-fidelity simulation consists of three phases: preparation (prebriefing), participation (simulation scenarios), and debriefing (Jimenez, 2021).

‘Experiential learning theory’ (Kolb, 2014) serves as the theoretical framework for this study, highlighting four essential phases: concrete experience, reflective observation, abstract conceptualization, and active experimentation. By ‘learning through experience,’ the learner actively engages in the learning process, with knowledge being gained through an ongoing cycle of questioning, reflecting, analyzing, and synthesizing. Students must actively construct meaning and conceptualization from their experience by critically reflecting on it both during and after it occurs. This framework is particularly relevant in simulation-based nursing education. Students engage in simulated scenarios that foster active participation and reflection, thus bridging the gap between theory and practice (Bresolin et al., 2022). During the debriefing phase, reflective observation encourages introspection. Abstract conceptualization emerges when students connect theory to practice, and active experimentation enables the application of knowledge in subsequent simulations or real clinical practice (Ross, 2021).

In current nursing practice, patient care adopts a holistic approach that considers all dimensions: biological, psychological, social, and spiritual (Öntürk Akyüz et al., 2024). The spiritual dimension is a critical component of holistic care, and nurses are expected to address patients' spiritual needs (Soto Morales et al., 2020). Accompanying patients' spiritual needs has been associated with improving the therapeutic relationship, fostering trust, and enhancing the overall quality of care (Puchalski, 2001; Sun et al., 2021). However, a recent meta-analysis revealed that nurses' competencies and perceptions regarding spirituality and spiritual care are moderate, highlighting the need for improvement (Wang et al., 2023). There are significant barriers to providing high-quality spiritual care, one of which is the lack of adequate training (Ross et al., 2022). Therefore, it is crucial to utilize effective educational tools for developing competencies in spiritual care. Research has shown that simulation is a promising method for teaching spiritual competencies (Connors et al., 2017; Huehn et al., 2019; Wang et al., 2023). Although further studies are needed to confirm its effectiveness across various contexts (Rykkje et al., 2022). To address this knowledge gap, we posed the following research question: *Is high-fidelity simulation effective in improving spiritual competencies among undergraduate nursing students?*

## Background

High-fidelity simulation has become increasingly prominent in nursing education, as it provides a realistic and immersive learning environment (Arrogante et al., 2023). This educational technique has been shown to enhance self-efficacy, confidence, and competencies among nursing students (Kim et al., 2016), also enabling them to achieve an optimal level of learning and develop critical, reflective thinking skills essential for knowledge generation (Cabero Almenara & Llorente Cejudo, 2015; Raurell-Torredà et al., 2020). It is recommended to incorporate high-fisssdelity simulation across all areas of nursing education (Filomeno & Minciullo, 2021).

In contemporary nursing practice, holistic patient care encompasses biological, psychological, social, and spiritual dimensions (Öntürk Akyüz et al., 2024). The spiritual aspect of care is essential in addressing the comprehensive needs of patients, and nurses play a key role in providing this type of care (Soto Morales et al., 2020). Numerous benefits are linked to spiritual health: improved ability to cope with the psychological and physical demands of illness, a reduction in pain, stress, and negative emotions, and a lower risk of depression and suicide. The data indicate that patients who receive the right kind of spiritual care are also more satisfied with the hospital's care and treatment (Harrad et al., 2019). Nonetheless, a recent meta-analysis found that nurses’ competencies in spiritual care are moderate, indicating a need for further training (Wang et al., 2023). One of the primary barriers to effective spiritual care is insufficient training (Ross et al., 2022). Given this challenge, simulation stands out as a promising tool for developing spiritual competencies, with prior studies demonstrating its effectiveness in teaching these skills (Connors et al., 2017; Huehn et al., 2019; Wang et al., 2023). However, additional research is needed to strengthen the evidence for its effectiveness in diverse educational contexts (Rykkje et al., 2022). Before conducting the current study, the SSCRS_Sp scale was validated with nursing students. The results of the questionnaire validation have been published in a separate article.

### Aim

The aim of this study was to implement an experiential learning activity based on high-fidelity simulation and evaluate its impact on nursing students' perceptions of increased training resources in spiritual care.

## Methods

### Development of the High-Fidelity Clinical Simulation Program

**Fig. 1:**
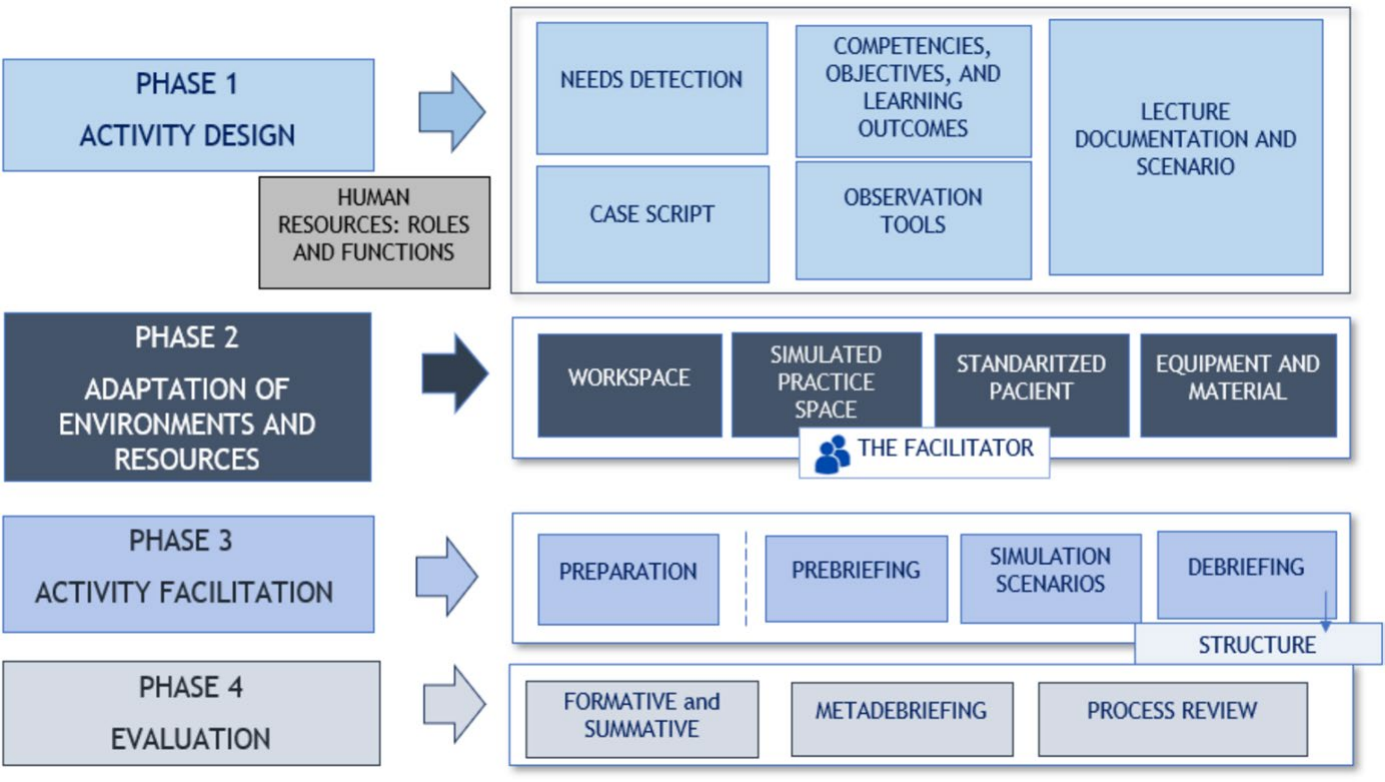
Phases of simulation activity according to Watts et al. (2021)

Following the standards described by the International Nursing Association for Clinical Simulation and Learning (INACSL) (Watts et al., 2021), a high-fidelity simulation program was designed and implemented with its development adhering to a structured and carefully planned timeline. Figure 1 outlines the phases of development for the high-fidelity simulation designed for this study. In phase 1 of the activity design, the students' learning needs in spiritual care were identified, and the desired objectives and learning outcomes were established. The scenarios were created by an expert in spiritual care (L.M).

The spiritual care needs were incorporated into the design of each simulated scenario with the aim of achieving the learning objectives established according to (INACSL, 2016) standards. The scenarios followed a chronological sequence centered on the same adult patient, admitted to a hospital ward, with spiritual needs requiring nursing care. In Phase 2 of the activity design, resources and the environment were prepared, including the selection of an actor as a standardized patient, the scenario, and the necessary materials. To represent the patient central to the case, a professional actor was used, as non-clinical skills are developed more effectively through interaction with a real actor rather than a mannequin. This actor had extensive experience in clinical simulation cases. In Phase 3 of the activity, the experts in spiritual care, along with the facilitator, conducted the various elements of the simulation: prebriefing, simulated scenarios, and debriefing. Finally, in Phase 4, the evaluation process of the activity was conducted.

For our study, we carried out the following learning activity based on (INACSL, 2016) (Table 1). In Phase 1, the students completed the pre-simulation questionnaire (SSCRS-Sp). In Phase 2, an expert educator in spiritual care, LM, conducted a preparatory session for the students before the simulation. In Phase 3 of the simulation, each class group, composed of (*n* = 40) students, was divided into smaller groups of (*n* = 10) students, with each (*n* = 2–3) students working through the simulated case according to each scenario. Each simulated scenario lasted 15 min, followed by a 30-min group debriefing with the medium-sized groups of (*n* = 10) students, led by an advanced practice nurse specializing in spiritual care (J.D). In Phase 4, the students answered the question: ‘*What does spirituality mean to you?’*. In Phase 5, the students completed the post-simulation questionnaire, SSCRS-Sp.

**Table 1:** High-fidelity spiritual simulation phases development of this study

Phase 1	Students answered questionnaire PRE (SCSSR-Sp)	
Phase 2	Participant preparation (introducing spirituality)	Two weeks before simulation students received a (2 h) theoretical master class on spirituality. Also, students were provided with the handouts of the simulation, covering a brief description of the simulation scenarios, readings and learning objectives
Phase 3	Simulation Prebriefing Simulated scenarios Debriefing	*Objectives* Assess spiritual needs in the context of the patient Identify the manifestations of independence and dependence of their spiritual need Be able to identify the three subdimensions of SECPAL in the patient's expressions Provide comprehensive care promoting patient autonomy
Phase 4	Answering a reflective question	The students answered the reflective question: ‘*What does spirituality mean to you?’*
Phase 5	Students answered questionnaire POST (SCSSR-Sp)	

### Evaluation of the high-fidelity clinical simulation program

#### Study design

A quantitative experimental study with a pretest–posttest single-group design was conducted to analyze the effectiveness of high-fidelity clinical simulation in improving spiritual competencies. Additionally, the students' perceptions of training resources in spiritual care were analyzed in relation to their religious beliefs. A qualitative component was added to the quantitative study with the aim of describing the conceptual significance of spirituality for students during their learning process. This research adhered to rigorous guidelines for nonrandomized designs, as outlined by the Transparent Reporting of Evaluations with Nonrandomized Designs (TREND) (Des Jarlais et al., 2004). The rigor of the qualitative research was ensured by adhering to the COREQ criteria (Tong et al., 2007) and maintaining the criteria of credibility, transferability, dependability, and confirmability (Cohen & Crabtree, 2006).

#### Sample

A non-probability sampling method with a convenience sample was used. The study included first-year undergraduate nursing students from a Spanish public university enrolled in the course ‘Evidence-Based Practice in Comprehensive Patient Care I’ (150 h, 6 European Credit Transfer and Accumulation System (ECTS) credits).

#### Data Collection and Instruments

Data collection was conducted via Microsoft Forms® on the university's platform. The students completed a sociodemographic questionnaire and the validated Spanish version for nursing students of the Spiritual Competence Scale, ‘Spirituality and Spiritual Care Assessment Scale’ (SSCRS-Sp) (Cronbach's alpha = 0.72), both before and after participating in the simulation. All documents were anonymized and coded for subsequent analysis. The SSCRS was developed by McSherry et al. (2002) based on a rigorous literature review and information obtained from 549 clinical nurses working in the National Health Service in England, addressing the need for a tool to measure nurses' understanding and attitudes toward spirituality and spiritual care. This scale was translated and validated into Spanish Fernández-Pascual et al. (2024) and consists of 17 items with a 5-point Likert scale response (0 = strongly disagree; 5 = strongly agree), grouped into four dimensions: Spirituality, Spiritual Care, Religiosity, and Personalized Care.

To identify and describe students' perceptions of training resources in spiritual care in relation to their religious beliefs, an ad hoc questionnaire was developed with the following four questions: *(1) Do you believe you have the necessary educational resources to address patients' spiritual needs? (2) Do you consider yourself a religious person? (3) Do you believe there is something after death? (4) What role or influence does religion have in your life?* Following the implementation of the questionnaires after the simulation, the students responded through an individual reflective narrative assignment to the question: ‘*What does spirituality mean to you?’.*

#### Data Analysis

Statistical analysis was performed using the statistical software R-4.4.1. The data are presented with the pre- and post-intervention means and standard deviations. To examine the data distribution before and after the intervention and to determine whether statistically significant differences existed, a series of hypothesis tests on the mean responses of the students were employed. Considering the sample size (*n* = 143) and according to the central limit theorem (Roca Martinez & Benítez López, 2023), it can be assumed that the underlying distribution of any statistic calculated with these data is approximately a Gaussian (normal) distribution. A paired-samples t-test was used to assess the effect of the intervention. In addition, the nonparametric Wilcoxon signed-rank test was calculated to corroborate the robustness of the conclusions drawn from the parametric tests and to examine whether both the parametric and nonparametric tests yielded the same conclusions.

Both types of tests reached the same statistical conclusions, providing greater robustness to the overall analysis. For both types of tests, a significance level of *p* < 0.05 was considered significant. In other words, a significance level (α) of 5% was considered for this study. Qualitative results were analyzed following Miller et al. (2012) recommendations and based on the students' responses to the question: *‘What does spirituality mean to you?’.* The lexicometric content analysis was conducted using the software IRAMUTEQ *(Interface de R pour les Analyses Multidimensionnelles de Textes et de Questionnaires)* version 0.7 alpha 2020. This program allows for textual analysis using qualitative algorithms based on the employed lexicon, utilizing the descending hierarchical classification described in Reinert's method (Morales del Río, 2019). This classification defines a series of lexical classes, each representing a theme that can be described by different words that help define it. The resulting images provide a graphical visualization that illustrates the intensity of relationships between words. For the development of this article, it was deemed inappropriate to provide a literal translation into English of the graphs used as examples.

#### Ethical considerations

This study was conducted in accordance with all ethical principles of the Helsinki Declaration, and the recommendations for quantitative research by Barker et al. (2024). Informed consent was obtained from all participants, and the confidentiality of the collected data was ensured (Lev et al., 2010). The Ethics Committee (CERT113) approved the study. Permission was obtained from the university's Data Protection Officer for the processing, management, and publication of the results.

## Results

The results are organized into four sections: (1) characteristics of participants; (2) effectiveness of simulation in improving students’ spiritual competencies; (3) relationship between religious beliefs and students’ perception of educational resources; and (4) conceptual significance of 'spirituality' for students.

### Characteristics of Participants

The total sample consisted of (*n* = 143) first-year undergraduate nursing students. The average age of the sample was 20.72 years. Regarding gender, 88% of the student sample were women and 11% were men. Additionally, 83.92% (*n* = 120) of the students identified as somewhat or not at all religious, while 16.09% (*n* = 23) considered themselves very religious. However, nearly 30% of the sample attributed a positive effect of religion in their lives, 55.2% reported no effect, 16.1% were unsure, and 1% attributed a negative effect. The sociodemographic characteristics of the students are summarized in Table 2.

**Table 2:** Descriptive characteristics of the participants (*n* = 143)

Students’ characteristics	*n*	(%)
*Gender*		
Female	126	88.11
Male	17	11.89
*Age*		
17–18	47	32.87
19	27	18.88
20	22	15.38
21–22	24	16.78
> 22	23	16.08
x̄ ± SD 20.72 ± 4.4		
*Civil status*		
Single	124	86.71
Married	4	(2.8
Live with someone	15	10.49
*Number of children*		
0	140	97.9
2	2	1.4
3	1	0.7
*Level of studies completed*		
High school	96	67.13
CGS	42	29.37
Other degrees	4	2.8
Master	1	0.7
*Students’ perception of their Spiritual care formative resources*		
None	5	3.5
Very few resources	82	57.34
Sufficient resources	34	23.78
Quite a few resources	21	14.69
Many resources	1	0.7
*Students’ perception of their own religiosity*		
Not religious at all	64	44.76
Slightly religious	56	39.16
Moderately religious	19	13.29
Very religious	4	2.08
*Students believe there is something after death*		
There is nothing	42	29.37
There is anything	53	37.06
I think so	31	21.68
Yes, sure	17	11.89
*Students’ perceptions of the role of region in their life*		
Any role	79	55.24
A positive influence	40	27.97
A negative influence	1	0.7
I do not know	23	16.08

### Effectiveness of Simulation in Improving Students’ Spiritual Competencies

Table 3 summarizes the results of the analysis of the efficacy of high-fidelity clinical simulation in improving spiritual competencies. The data are presented with the pre- and post-intervention means and standard deviations for each of the 17 items on the scale data. Analysis revealed statistically significant differences for items d, e, and *n* (*p* < 0.05); c, g, j, k, and l (*p* < 0.01); and a, c, f, h, i, o, and q (*p* < 0.001).

**Table 3:** Pretest–posttest results for the SSCRS-Sp scale

Question	Pre x̅ (SD)	Post x̅ (SD)	Paired *t*-test (statistic)	Paired *t*-test (*p*-value)	Wilcoxon signed-rank test (statistic)	Wilcoxon signed-rank test (*p*-value)	Signification
(a) I believe that nursing staff can provide spiritual care by arranging meetings with the hospital chaplain, priest, pastor, rabbi, or the patient’s spiritual director if requested	3.64 (0.817)	3.99 (0.809)	− 4.2643	1.819e − 05	872	1.899e − 05	***
(b) I believe that nursing staff can provide spiritual care by demonstrating kindness, concern, and empathy	4.57 (0.588)	4.6 (0.546)	− 0.5684	0.2853	677	0.3541	
(c) I believe that spirituality is related to the need to forgive and be forgiven	3.39 (0.935)	4.04 (0.804)	− 7.3607	6.757e − 12	684	1.874e − 10	***
(d) I believe that spirituality solely involves attending church or a place of religious worship	4.28 (0.851)	4.45 (0.836)	− 1.9274	0.02796	1082	0.01132	*
(e) I believe that spirituality is not related to belief and faith in God or a higher being	3.1 (1.06)	3.33 (1.08)	− 2.1655	0.01601	1372	0.01916	*
(f) I believe that spirituality involves seeking meaning in both the good and bad situations in life	3.96 (0.701)	4.26 (0.668)	− 4.6187	4.287e − 06	529	7.347e − 06	***
(g) I believe that nursing staff can provide spiritual care by accompanying the patient, offering support, encouragement, and strength, especially in times of need	4.54 (0.602)	4.68 (0.525)	− 2.5069	0.006653	392	0.007585	**
(h) I believe that nursing staff can provide spiritual care by allowing the patient to find meaning and purpose in their illness	3.97 (0.839)	4.36 (0.717)	− 4.9311	1.126e − 06	542.5	1.06e − 06	***
(i) I believe that spirituality involves having a sense of hope in life	3.74 (0.845)	4.15 (0.75)	− 5.2657	2.531e − 07	620	5.274e − 07	***
(j) I believe that spirituality is related to how one lives their life in the here and now	3.99 (0.796)	4.2 (0.753)	− 2.8285	0.00268	954.5	0.00351	**
(k) I believe that nursing staff can provide spiritual care by listening to patients and dedicating time to talk, exploring their fears, anxieties, and problems	4.44 (0.657)	4.62 (0.556)	− 3.052	0.00136	532	0.00149	**
(l) I believe that spirituality is a unifying force that allows one to be at peace with oneself and the world	4.22 (0.686)	4.41 (0.62)	− 3.1298	0.00106	524	0.001274	**
(m) I believe that spirituality does not include aspects such as art, creativity, and self-expression	3.85 (0.937)	3.61 (1.21)	2.1097	0.9817	2303	0.9845	
(n) I believe that nursing staff can provide spiritual care by respecting the patient’s privacy, dignity, and religious and cultural beliefs	4.42 (0.654)	4.56 (0.564)	− 2.2682	0.01241	721	0.01413	*
(o) I believe that spirituality includes friendships and social relationships	3.8 (0.771)	4.34 (0.712)	− 7.5132	2.946e − 12	343	1.203e − 10	***
(p) I believe that spirituality is not possible for atheists (those who deny or do not believe in the existence of God) or agnostics (those who are uncertain about the existence of God)	4.34 (0.733)	4.42 (0.915)	− 0.9464	0.1728	798.5	0.09217	
(q) I believe that spirituality is related to a person’s morality	3.83 (0.725)	4.07 (0.747)	− 3.3189	0.000574	785	0.000740	***

* *p* < 0.05; ** *p* < 0.01; *** *p* < 0.001

Table 4 presents the results based on the grouping of items from the SSCRS_Sp: (I) Spirituality (items: f, h, i, j, l); (II) Spiritual Care (items: a, b, g, k, *n*); (III) Religiosity (items: d, m, *p*); and (IV) Personalized Care (items: *n*, o, q).

**Table 4:** Pretest–posttest results by item groups

Item	Pre *x̅* (SD)	Post *x̅* (SD)	Paired *t*-test (statistic)	Paired *t*-test (*p*-value)	Wilcoxon signed-rank test (statistic)	Wilcoxon signed-rank test (*p*-value)	Signification
(I) Spirituality (items f, h, i, j, l)	19.9 (2.62)	21.4 (2.57)	−6.7921	1.399e−10	1206	1.913e−09	***
(II) Spiritual Care (items a, b, g, k, n)	21.6 (2.09)	22.4 (2.11)	−4.5219	6.41e−06	1953	1.814e−05	***
(III) Religiosity (items d, m, *p*)	12.5 (1.87)	12.5 (2.33)	−0.0346	0.4862	2755	0.3366	
(IV) Personalized Care (items n, o, q)	12 (1.54)	13 (1.49)	−6.6358	3.155e−10	1182	3.922e−09	***

* *p* < 0.05; ** *p* < 0.01; *** *p* < 0.001

Results for the items in group (I) ‘Spirituality’ indicated that students, after the simulation, improved their perception that spirituality is not solely a concept associated with a religion, belief system, or worship, but has a universal character that acknowledges areas such as creativity, art, and self-expression (*p* < 0.001).

Results for the items in group (II) ‘Spiritual Care’ indicated that students improved their perception that spiritual care is related to nurses providing care that includes listening, maintaining privacy and dignity, promoting the discovery of personal meaning and purpose, and demonstrating attitudes such as kindness, concern, and empathy (*p* < 0.001).

Regarding group (III) ‘Religiosity,’ the results indicated that there were no significant differences in students' perceptions of their own religious beliefs after the simulation (*p* = 0.4862). Students continued to express that religiosity is an important component of spirituality, but that individuals may or may not express their spirituality through it.

Regarding group (IV) ‘Personalized Care,’ a statistically significant difference was observed between students' scores before and after the simulation (*p* < 0.001). After completing the simulation, students were much more in agreement that all dimensions of spirituality and life are unique to each individual. Additionally, they improved their perception that nurses can provide spiritual care while respecting the patient's privacy, dignity, and religious and cultural beliefs.

### Relationship Between Religious Beliefs and Students’ Perception of Educational Resources

Table 5 presents the results of the analysis of students' perceptions of educational resources in spiritual care in relation to their religious beliefs.

**Table 5:** Students' perception of educational resources in spiritual care in relation to their religious beliefs

Question	Pre *x̅* (SD)	Post *x̅* (SD)	Paired *t*-test (statistic)	Paired *t*-test (*p*-value)	Wilcoxon signed-rank test (statistic)	Wilcoxon signed-rank test (*p*-value)	Signification
1. Do you believe you have the necessary educational resources to address patients' spiritual needs?	2.52 (0.81)	3.2 (0.81)	− 7.6528	1.369e − 12	768	5.72e − 11	***
2. Do you believe there is something after death?	2.16 (0.98)	2.29 (1.02)	− 2.0129	0.02301	415	0.03019	*
3. Do you consider yourself a religious person?	1.74 (0.79)	1.74 (0.80)	0	0.5	195	0.5688	
4. What role or influence does religion have in your life?	1.78 (1.08)	1.78 (1.1)	0	0.5	236.5	0.5377	

*p < 0.05; **p < 0.01; ***p < 0.001

A pre- and post-intervention statistical analysis was conducted on the responses to the following questions: (1) *Do you believe you have the necessary educational resources to address patients' spiritual needs? *(2)* Do you consider yourself a religious person? *(3)* Do you believe that something exists after death? *(4) *What role or influence does religion have in your life?*

Students perceived that their educational resources regarding spiritual care had significantly increased after the simulation (*p* < 0.001), regardless of their religious beliefs. The results indicated that participating in the high-fidelity simulation on spirituality did not alter students' religious beliefs (*p* = 0.5). It is noteworthy that students who identified as very religious also felt they had sufficient educational resources both before and after the simulation. However, these results should be interpreted with caution, as they are descriptive in nature and their statistical significance was not tested due to the small number of students (*n* = 4) in this subgroup relative to the total sample.

### Conceptual Significance of 'Spirituality' for Students

**Fig. 2:**
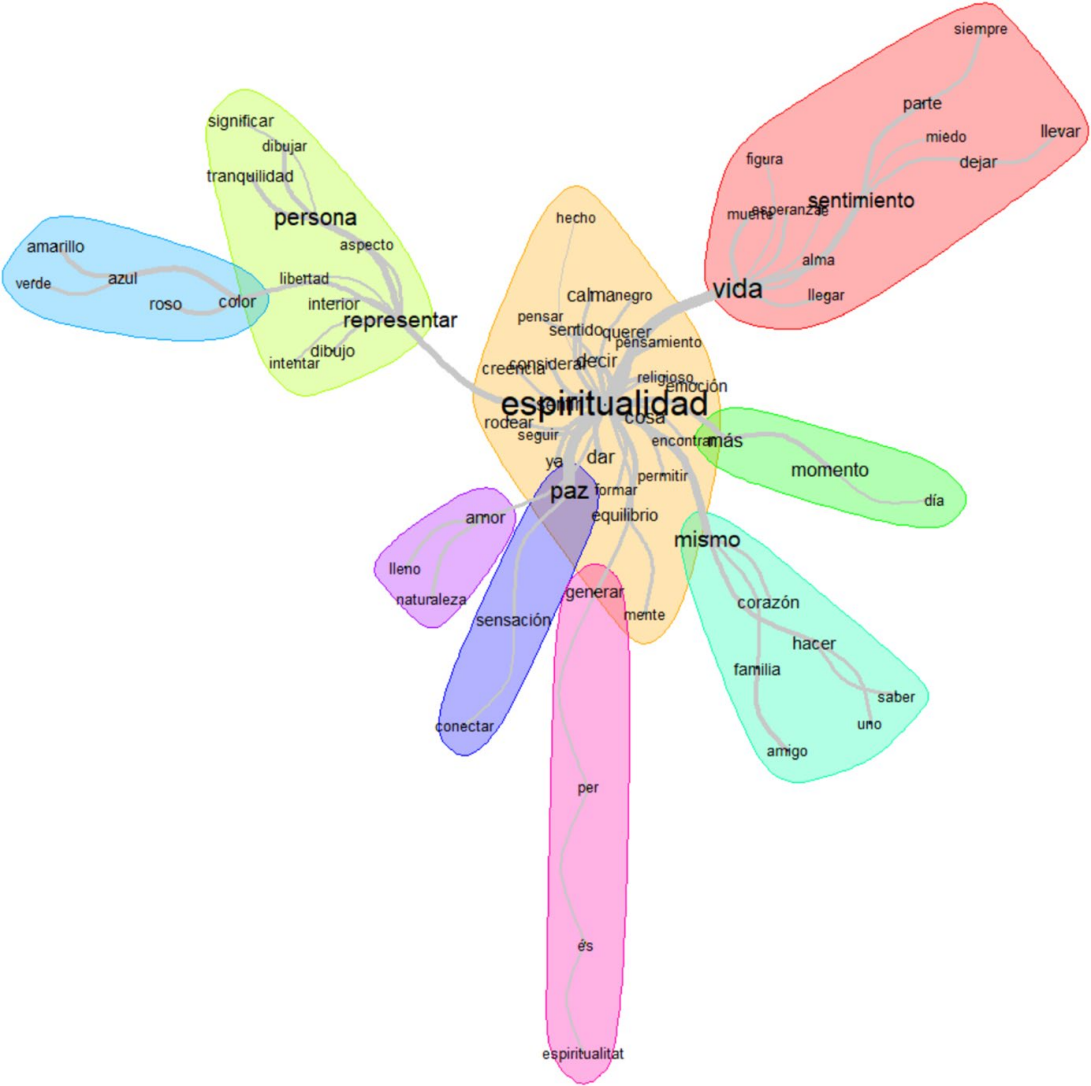
IRAMUTEQ figure on the conceptual meaning of 'spirituality' for students

Figure 2 graphically represents the lexicometric analysis of students' responses regarding the conceptual significance of spirituality during their learning. Table 6 summarizes the results of the thematic analysis of students' responses to the question: *‘What does spirituality mean to you?’.* The words with the highest frequencies are: (1) Spirituality (frequency 36), as it is the focal term of the question; (2) Nouns: life (frequency 24), person (frequency 20), peace (frequency 17), feeling (frequency 13), moment (frequency 13); (3) Verbs: represent (frequency 18), feel (frequency 11), and give (frequency 11); and (4) Adverb: more (frequency 11).

**Table 6:** Results of the analysis of students' responses to the question: *What does spirituality mean to you?*

Concept	Relationship with Spirituality	Related words	Participants quotes
Life	It is part of human life	Feeling Always Fear Death	S14: ‘way of being, experiences, the influence of your environment. But at the same time, they are your beliefs, your feelings’ S5: ‘helps us to escape the feeling of uncertainty and fear that death provokes in us’ S3: ‘Spirituality alleviates fear and doubt and allows us to move forward without focusing on the bleak truth’
Representations	It may encompass various subjective meanings for each individual	Freedom Love Peaceful Soul	S2: ‘the love you can receive, even the love you can give’ S5: ‘the power to choose your path in life’ S17: ‘the feeling of floating on clouds of being free’
Peace	It refers to the feeling of spirituality	Love Nature Sensation Connection	S3: ‘when I am at night in the mountains looking at the stars and reflecting’ S5: ‘it's that inner peace achieved through connection with myself’ S8: ‘being in harmony with all the elements of the Earth, feeling peace’ S14: ‘nature and the connection with animals’
Finding	It relates to self-knowledge and relationships with others, exploring the depths of the heart	Self Family Friend Heart	S8: ‘warm things from my day that make me feel fulfilled: love for family, my friends, nature, and myself (heart within a heart)’ S10: ‘family, the ability to choose your path in life’

Qualitative results indicated that for students, the concept of spirituality is shaped by life, as a feeling of surrendering, sometimes with a sense of fear, in order to reach the soul and hope. Students described a person's spirituality as rooted in freedom, love, and tranquility. When providing spiritual care to patients, they also highlighted ‘learning to find oneself’ as a significant aspect of spirituality, which reflects its intrapersonal dimension.

## Discussion

This research implemented and evaluated a high-fidelity simulation intervention in spiritual care for nursing students.

The results indicate that this simulation could be an effective learning activity for improving students' competencies in spiritual care. Our results align with the findings of the study by Connors et al. (2017), where students significantly increased their competence and confidence in addressing patients' spiritual care through simulation. In the same vein, the study by Huehn et al. (2019) also determined that students found the learning experience through simulation beneficial for their preparation to provide spiritual care in professional practice.

Other learning activities (case discussions, reflective practice journals, or lectures) have shown positive results regarding the learning of competencies in spiritual care. Although the recent review by Rykkje et al. (2022) suggests the use of pedagogical and reflective learning strategies allow students to apply what they have learned in the classroom and reflect on it.

Based on Kolb's (2014) experiential learning theory, which emphasizes the integration of learning through four phases (concrete experience, reflective observation, abstract conceptualization, and active experimentation) to demonstrate how individuals learn and master their experiences, our students were able to reflect on the concept of spirituality based on their experiences. The debriefings conducted after the simulation facilitated a deeper integration of their learning.

This learning objective of developing spiritual competencies through experience and reflection is also echoed by students in various studies (Bresolin et al., 2022; Ross, 2021). Additionally, the theoretical learning of spiritual care, which begins with the student's awareness of spirituality (Fernández-Pascual et al., 2020), is reinforced by the simulation activity. Particularly, through the subsequent debriefing that fosters both personal and group reflection among students.

Based on our results and those detailed in other studies, simulation solidifies its role as a highly effective learning activity for stimulating students' acquisition of spiritual competencies (Rykkje et al., 2022). On the other hand, one of the advantages of simulation is that it is an educational strategy that can be implemented throughout the nursing degree program, during students' clinical placements, and throughout their professional careers (Jones et al., 2022). This advantage would address the limitation of some learning activities regarding their long-term effects (Daudt et al., 2019). It would be advisable to regularly implement simulation activities in nursing degree and in the ongoing training of nurses, while evaluating their effectiveness longitudinally.

Although our results regarding the positive relationship between students' religious beliefs and their perception of their educational resources should be interpreted with caution, various studies indicate that spirituality is influenced by these beliefs (Fernández-Pascual et al., 2020). This conditioning is also emphasized by Torres-Contreras et al. (2022), who additionally highlight the impact of the student's cultural and geographical context. In this regard, students from Hindu, Latin, or Buddhist cultures perceive that they have greater educational resources for providing spiritual care (Al Qadire et al., 2024).

In the recent meta-analysis by Wang et al. (2023), it was identified that nurses with religious affiliations demonstrated stronger spiritual care competencies compared to nurses without religious affiliations. However, after simulation, our students agree more strongly that spirituality extends beyond being synonymous with religion. Students stated as it encompasses universal aspects such as creativity, art, and self-expression. It is important to emphasize this shift in perception among students, as nurses have identified the persistent misconception that spirituality is solely synonymous with religion as a significant barrier to addressing spirituality in practice (Ross et al., 2018).

## Limitations

This study was conducted using a single-group, cross-sectional design, which limits the ability to establish causality and assess long-term knowledge retention (White & Arzi, 2005). While the inclusion of a qualitative component provided valuable narrative and descriptive insights, the absence of a control group reduces the robustness of the findings. Future studies should consider employing quasi-experimental designs with a control group to enhance the validity of the results and assess the intervention's impact more effectively (Barker et al., 2024). Another limitation is the sample size, which, although sufficient for the scope of this exploratory study, may not be large enough to generalize the findings to a broader population. Larger sample sizes in future research would increase statistical power and improve the generalizability of the results across diverse educational and cultural contexts. Additionally, potential biases related to participants' self-selection and the use of self-reported measures must be considered, as these may have influenced the reported outcomes.

Implementing randomized sampling and incorporating objective assessments in future studies could help mitigate these biases. The study was also limited by its focus on a single institution, which may affect the transferability of the findings to other nursing programs or educational settings. To address this, future research should adopt international and multicenter approaches, which would provide a more diverse sample and allow for cross-cultural comparisons. Finally, the study did not assess the integration of acquired competencies into students' clinical practice. Future research should investigate how simulation-based learning in spiritual care influences students’ confidence and competence in real clinical settings, and whether these skills are retained and effectively applied over time.

### Implications for Education and Research

This research highlights the potential of high-fidelity clinical simulation as a learning activity for improving spiritual care competencies in nursing students. Our results provide valuable insights for incorporating new, engaging, and reflective teaching strategies for learning about spiritual care in nursing education. Simulation in spiritual competencies has proven to be an educational and reflective resource adaptable to each context. Awareness of the importance of spiritual care should be continuously emphasized, and there must be moments and opportunities in practice to dedicate time to the spiritual care of individuals. Finally, nursing managers and educators bear the responsibility of planning and implementing ongoing training in spiritual care, tailored to each context, as part of the broader goal of improving and humanizing nursing care.

## Conclusions

High-fidelity clinical simulation has proven useful in improving students' educational resources for providing spiritual care. Additionally, through this activity, students understood spirituality as an essential part of holistic nursing care, something subjective that relates to feelings of peace, freedom, and one's perspective on life. On the other hand, students became aware that spirituality extends beyond religion, although it may be a part of it. To explore the perspectives and experiences of nursing students regarding simulation and spiritual care, a qualitative study has been conducted. The results of this qualitative study will be published in a subsequent article.
